# Supplementary material for: A Quick Method to Synthesize Extrachromosomal Circular DNA In Vitro
Source: Molecules. 2023 May 22;28(10):4236. doi: 10.3390/molecules28104236 (PMC10220718; doi:10.3390/molecules28104236)
Supplement: Supplementary file 1 [file molecules-28-04236-s001.zip › molecules-2308311-supplementary.pdf]

A Quick Method to Synthesize Extrachromosomal Circular DNA In Vitro

Shanru Zuo [1,2,†], Xueguang Li [1,†], Yide Yang [1], Junhua Zhou [1] and Quanyuan He [1,*]

Supplementary Materials

Figures

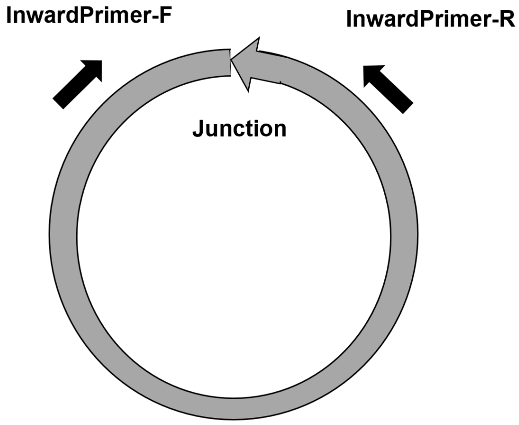

Figure S1. Schematic illustration of the inward PCR primer design.

Tables

Table S1. The information of the used primers in the study.

Primer name	sequence(5'→3')
2392-1F	CCACAGAAATGTGGACCTGG
2392-1R	GGCCTTCTAATGGTATCTTAAGG
2392-2F	GAAATGTGGACCTGGAAGGG
2392-2R	TGTGGGGCCTTCTAATGGTATCTTAAGG
2392-3F	CATAGTGAAGTCTATGCCGTAC
2392-3R	CAAATGAGTGATGATTTCCGC
BRCA1-1F	GAGTCAGTCACATGGACTTAAC
BRCA1-1R	GACTGACTCAGTCTGCCAAAAG
BRCA1-2F	CAGTCACATGGACTTAACAATAATG
BRCA1-2R	ACTCGACTGACTCAGTCTGCC
BRCA1-3F	CTGTTTTCTCTTAGTTGGCCCAC
BRCA1-3R	GAAGTGGGAGATTCTAGCTTAG
LIMD1-1F	TGCTGGATGTGTGCAGCTTATG
LIMD1-1R	GCCCAGCAGAGTAGGGAAAG
LIMD1-2F	GGATGTGTGCAGCTTATGGTAG
LIMD1-2R	AGCAGCCCAGCAGAGTAGGGAAAG
LIMD1-3F	GGACTTGAACCTGACAGTTCAGTTC
LIMD1-3R	CAGCAGTACCCAATATGACCTGTG

Table S2. The information of the used inward-PCR primers in the study.

Primer name	sequence(5'→3')
Inward-BRCA1-F	CTGTTTTCTCTTAGTTGGCCCAC
Inward-BRCA1-R	GAAGTGGGAGATTCTAGCTTAG
Inward-2392F	CCATCCTACAGCCTGGGGTG
Inward-2392R	CATTTCTCCTGGCCCAATCACTC
Inward-LIMD1-F	GTCTCAAGATGCCCAGTTTC
Inward-LIMD1- R	CAACAAGGCAAGACCTTGTCTC

Table S3. The detailed protocol of the QuickLAMA.

Procedure	Reaction System	Condition	Reagent (Company, CatNo.)	Description	Time
Step 1	2*PCR buffer for KODFX 25μl<br>2mMdNTPs 10μl<br>Primer-F(10pmol/μl) 1.5μl<br>Primer-R(10pmol/μl) 1.5μl<br>genomic-DNA x μl<br>PCR grade water y μl<br>KODFX 1ul<br>Total volume 50ul	Pre-denature 94℃ 2min<br>Denature 98℃ 10sec<br>Annealing (Tm-5)℃ 30sec<br>Extension 68℃ 1kb/min<br>(Denature–Extension: 20~30 cycles)<br>Hold 4℃ ∞	KODFX (TOYOBO, KFX-101)	PCR to amply the target eccDNA region from the genome to synthesize the fragment A. And using this system to synthesize the C-long, C-short, D-short and D-long fragments (see Figure 2)	1-3h
Step 2	short DNA 20μl<br>long DNA 20μl<br>5*DNA Annealing buffer 10μl<br>Total volume 50μl	Pre-denature 94℃ 5min<br>Denature -5℃ 3min<br>Annealing 30℃ 3min<br>Hold 4℃ ∞	Annealing Buffer for DNA Oligo (Solarbio, D2810)	Denature and anneal the C-long, C-short, D-short and D-long fragments and obtain the hybrid fragments with one stick end.	50min
Step 3	DNA ligase reaction buffer 10μl<br>Sticky C 4.5μl<br>Sticky D 4.5μl<br>T7 DNA Ligase 1μl<br>Total volume 20μl	25℃ 0.5h<br>inactivation : 65℃ 10min	T7 DNA Ligase (NEB,M0318S)	Ligation of the fragments to form the fragment E as the template for the next step.	40min

Step 4	2*PCR buffer for KODFX 25μl<br>2mMdNTPs 10μl<br>Primer-3F(10pmol/μl) 1.5μl<br>Primer-3R(10pmol/μl) 1.5μl<br>Step3 product (template) x μl<br>PCR grade water y μl<br>KODFX 1μl<br>Total volume 50μl	Predenature 94℃ 2min<br>Denature 98℃ 10sec<br>Annealing (Tm-5)℃ 30sec<br>Extension 68℃ 1kb/min<br>(Denature–Extension: 15~30 cycles)<br>Hold 4℃ ∞	KODFX (TOYOBO, KFX-101)	PCR to amply the fragment E. Notice: the Step3 product usually needs to be diluted to avoid adding too much template.	40min-2h
Step 5	DNA-A/E 39μl<br>T4 PNK reaction buffer 5μl<br>ATP 5μl<br>T4 PNK 1μl<br>Total volume 50μl	37℃ 30min<br>inactive condition: 65℃20min	T4 PNK (NEB, M0201S)	Add a phosphate group to the fragment A and E	1h
Step 6	A-P' 22μl<br>E-P' 22μl<br>10*HIFI Taq DNA<br>Ligase buffer 5μl<br>HIFI Taq DNA Ligase 1μl<br>Total volume 50μl	Denature 95℃ 20sec<br>annealing 4℃ 1min<br>ligation 65℃ 20min<br>(3 cycles)	HIFI Taq DNA Ligase (NEB, M0647)	LAMA reaction for circularization and nick ligation	1h20min
Step 7	eccDNA(step6) 34μl<br>25mMATP 4μl<br>10*reaction Buffer 10μl<br>Plasmid-Safe DNase 2μl<br>ddH2O 50μl<br>Total volume 100μl	37℃ 1h<br>inactive condition: 70℃ 30min	Plasmid-Safe ATP-Dependent Dnase	Digest the remained linear DNA	1.5h

			(LGCBiosearch, E3101K)		
Step 8	Follow the kit's manual		E.Z.N.A. Cycle-Pure Kit (omega, D6492-02)	Purify the eccDNA /minicircle	20min
